# Trends in beliefs about the harmfulness and use of stop-smoking medications and smokeless tobacco products among cigarettes smokers: Findings from the ITC four-country survey

**DOI:** 10.1186/1477-7517-8-21

**Published:** 2011-08-23

**Authors:** Ron Borland, Jae Cooper, Ann McNeill, Richard O'Connor, K Michael Cummings

**Affiliations:** 1 VicHealth Center for Tobacco Control, The Cancer Council Victoria, 1 Rathdowne St, Carlton 3053, Victoria, Australia; 2 UK Centre for Tobacco Control Studies, Division of Epidemiology & Public Health, University of Nottingham, Nottingham NG51PB, UK; 3 Department of Health Behavior, Roswell Park Cancer Institute, Elm and Carlton Streets, Buffalo, NY 14263, USA

## Abstract

**Background:**

Evidence shows that smokers are generally misinformed about the relative harmfulness of nicotine, and smokeless forms of nicotine delivery in relation to smoked tobacco. This study explores changing trends in the beliefs about the harmfulness and use of stop smoking medications and smokeless tobacco in adult smokers in four countries where public education and access to alternative forms of nicotine is varied (Canada, the US, the UK and Australia).

**Methods:**

Data are from seven waves of the ITC-4 country study conducted between 2002 and 2009 with adult smokers from Canada, the US, the UK and Australia. For the purposes of this study, data were collected from 21,207 current smokers. Using generalised estimating equations to control for multiple response sets, multivariate models were tested to look for main effects of country, and trends across time, controlling for demographic variables.

**Results:**

Knowledge remained low in all countries, although UK smokers tended to be better informed. There was a small but significant improvement across time in the UK, but mixed effects in the other three countries. At the final wave, between 37.5% (US) and 61.4% (UK) reported that NRT is a lot less harmful than cigarettes. In Canada and the US, where smokeless tobacco is marketed, only around one in six believed some smokeless tobacco products could be less harmful than cigarettes.

**Conclusions:**

Many smokers continue to be misinformed about the relative safety of nicotine and alternatives to smoked tobacco, especially in the US and Canada. Concerted efforts to educate UK smokers have probably improved their knowledge. Further research is required to assess whether misinformation deters smokers from appropriate use of alternative forms of nicotine.

## Background

Most smokers have tried to quit, and many try repeatedly without success. Providing alternatives in the form of nicotine replacement therapy (NRT) has been shown to facilitate long-term cessation [1]. Smokers should be properly informed about ways they can reduce their risks of harm [2]. As far as we know there are no serious health effects of use of NRT to quit (except perhaps during pregnancy). As a result, NRT is increasingly available over the counter outside of pharmacies. The limited available evidence also shows that use of nicotine replacement products for up to at least 5 years is safe [3]. Evidence from use of the lowest toxin forms of smokeless tobacco (SLT) suggests that even longer use can be done with much lower risks compared to smoking [4]. The available evidence shows that nicotine is not a carcinogen [5], although it may be a co-factor in the cause of cancer [6].

Tobacco products are on the whole more harmful than pure nicotine as they contain other toxins and in the case of smoked products are taken into the lungs which is more sensitive tissue than the stomach (or skin in the case of nicotine patches). Typically, smokeless forms of tobacco are less harmful than smoked forms and there exist low toxin forms that produce few of the adverse effects of other tobacco products [7]. While many existing smokeless products are very harmful (e.g., South-East Asian and Sudanese forms; [8]), low nitrosamine versions like Swedish snus have been estimated to be 90 - 95% less harmful than cigarettes when used long-term [4], and others contend it is even less harmful [9]. There is no doubt that the toxicity of SLT can be systematically reduced without it unduly reducing user acceptability, something that has not been achieved for smoked tobacco. In Sweden, more ex-smokers report having quit using SLT than NRT, including some who continue to use it as a long-term substitute [10,11] and recent studies in Norway report similar findings [12,13]. SLT is not available in some Western countries, being banned in Australia and New Zealand and all European Union countries other than Sweden. It has remained available in the US and Canada. Despite this, most smokers are misinformed about the safety and efficacy of both NRT and SLT. For example, one study [14] found that a majority of US smokers erroneously believed that nicotine is a cause of cancer, while another found a large minority in four countries (US, UK, Australia and Canada) held the same misbelief in 2002, with it more prevalent among low socioeconomic status smokers [15]. The misinformation may be a barrier to use of it as an aid to quit smoking, or for premature discontinuation. O'Connor and colleagues [16] reported that less than 20% of smokers in Canada, the US, the UK and Australia believe that *any *smokeless products are less harmful than cigarettes, though this analysis appears to have underestimated knowledge, particularly in the UK and Australia. Even in Sweden, where SLT use is higher than smoked tobacco among males [17], a recent study has shown that Swedish cigarette smokers are misinformed about the relative safety of SLT [18].

The facts about relative harms and smokers lack of knowledge on this has gained some public exposure [e.g., [19,20]], so it is of interest to see whether there has been any improvement in smokers knowledge. The country where improvements in knowledge might be most likely is the UK. The Royal College of Physicians published two high profile reports, one on nicotine addiction and smoking in 2000 [21], and the other in 2007 [9] focusing on nicotine addiction and harm reduction. Both reports received public coverage about the role of nicotine in smoking and the second report in particular explored the role that different forms of nicotine delivery, including nicotine replacement therapies and low nitrosamine SLT products, could play in a harm reduction strategy. Based in part on this knowledge base, the UK smoking cessation strategy has involved training a national cadre of stop smoking advisors and specialists, from a variety of health professional backgrounds, to give advice and support to smokers wishing to quit [22]. Typically it has involved increasing knowledge about nicotine dependence and relative harms of NRT compared with smoking. Stop smoking advisors and specialists are also trained to interact with primary care professionals to enhance their knowledge and increase referrals to stop smoking services. The UK also changed it's licensing requirements for nicotine replacement medications permitting them to be given to pregnant women and labelled for used as a substitute for smoking [23]. So the message about stop-smoking medication not being harmful to health is one that is likely to be widely promulgated to UK smokers. There have also been a lot of mass media campaigns around NRT from pharmaceutical companies and some governmental campaigns that may also have helped to profile these messages.

The aim of this paper is to assess any trends in beliefs about the harmfulness of nicotine itself, stop-smoking medication including NRT, and SLT over the last 4 to 7 years in Canada, the US, the UK and Australia. This paper also examines the extent to which the beliefs vary by sociodemographic group, and how beliefs about nicotine related to use of NRT and SLT products.

## Method

### Data collection and sample

The ITC-4 is an annual survey conducted via computer-assisted telephone interview in Canada, UK, USA, and Australia. Respondents are selected via random-digit dialling to ensure a broadly representative sample. All respondents are smokers at the time of recruitment (smoked at least 100 cigarettes in their lifetime and smoked at least once in the past 30 days) but are retained at follow-up surveys if they quit smoking. At each wave, approximately 30% of the sample is replenished from the original sampling frame. A detailed description of the ITC project's conceptual framework [24] and methodology [25] can be found elsewhere. For this study, we selected respondents who were current smokers (daily, weekly, or monthly) at the time of each of the seven ITC-4 waves (2002 to 2008). Table 1 shows the number of eligible respondents at each baseline survey, and the distribution by demographic characteristics. Demographic trends remained fairly stable across the survey waves, although the sample was significantly older and of higher socioeconomic status (SES) at wave 7 compared to wave 1.

**Table 1:** (%; weighted)

	Wave 1 Nov - Dec 2002 n = 8930	Wave 2 May - Sept 2003 n = 7802	Wave 3 Jun - Dec 2004 n = 7503	Wave 4 Oct '05 - Jan '06 n = 7018	Wave 5 Oct '06 - Feb '07 n = 7038	Wave 6 Sept '07 - Feb '08 n = 6886	Wave 7 Oct '08 - Jun '09 n = 5886
Country							
Canada	24.5	25.7	25.1	25.1	24.6	24.7	25.7
US	23.6	24.3	25.6	25.5	25.4	25.1	22.6
UK	26.5	24.7	24.6	24.8	24.4	24.2	24.3
Australia	25.4	25.3	24.7	24.6	25.6	25.9	27.3
SES							
Low	24.3	23.0	21.9	23.4	23.4	20.7	21.5
Moderate	56.5	56.7	56.0	52.9	52.7	53.0	50.5
High	19.2	20.3	22.0	23.7	24.0	26.4	28.0
Gender							
Female	46.7	47.2	46.9	46.9	47.6	46.8	46.1
Age							
18 to 24	15.1	15.1	14.4	13.7	12.9	12.0	8.9
25 to 39	33.4	32.3	32.0	32.7	33.6	33.2	30.9
40 to 54	32.8	33.9	34.9	34.9	34.3	34.9	38.1
55 +	18.6	18.8	18.7	18.7	19.2	19.9	22.0

NB: SES = Socioeconomic Status.

### Measures

#### Main outcome measures

##### Beliefs about the safety of nicotine and alternatives to smoked tobacco

To assess knowledge of the relative harm of SLT respondents were asked, "Are you aware of any smokeless tobacco products, such as snuff or chewing tobacco, which are not burned or smoked but instead are usually put in the mouth?" Those who said yes were asked, "As far as you know, are ANY smokeless tobacco products less harmful than ordinary cigarettes?" Those who answered "yes" were asked whether they are a lot less harmful or less harmful. Respondents who answered "no" were asked whether they are more harmful or the same. Two measures were created with 1) "Less harmful" vs. "All other responses," and 2) "A LOT less harmful" vs. "All other responses". Because there is a wide range of SLT forms, and we did not explore precisely what product respondents were considering in giving their answer, we considered both 'less harmful' and 'a lot less harmful' to be correct answers. Due to an error in the survey, a substantial number of respondents were not asked this question at wave 4, and as such we do not report data for the wave 4 survey.

To assess knowledge of the harmfulness of NRT compared to smoked tobacco respondents were asked, "As far as you know, are nicotine replacement medications less harmful than smoking cigarettes?" Those who said "yes" were asked whether they are a lot less harmful or less harmful. Respondents who said "no" were asked whether they are more harmful or the same. A dichotomous measure was created with the correct belief "Lot less harmful" vs. "Little less harmful/same/more harmful/don't know". The correct answer is a lot less harmful.

At each wave beliefs about the harmfulness of stop-smoking medication were assessed by asking respondents to indicate on a five-point scale whether they 1) strongly agree through to 5) strongly disagree with the statement "Stop-smoking medications might harm your health". A dichotomous measure was created with "Agree/neither agree nor disagree/don't know" vs. "Disagree", with the latter treated as the appropriate answer

At each wave knowledge about the cancer risk posed by nicotine was assessed by asking respondents whether the statement "The nicotine in cigarettes is the chemical that causes most of the cancer" was true or false. The correct answer is false.

##### Recent use of any stop-smoking medication and NRT

At waves 1 and 2, respondents were asked whether they had used any stop-smoking medications in the previous 6 months (Yes or No). From wave 3 onwards, they were asked about this in reference to the last survey (or last 12 months for new recruits). To assess use of NRT specifically, respondents were then asked, "The last time you used medications *to quit smoking*, which product or combination of products did you use?" Respondents were read a list of current products available, including NRT and non-NRT prescription medication, and asked to indicate which one/s applied. A dichotomous measure was created with "Used NRT" vs. "Other medication or none at all".

##### Recent use of smokeless tobacco

At waves 1 and 2, respondents who were aware of SLT products were asked whether they had used any SLT in the previous 6 months (Yes or No). From wave 3 onwards, they were asked about this in reference to the last survey (or last 12 months for new recruits).

##### Demographics

Demographic variables included: age (18 - 24, 25 - 39, 40 - 54, & 55+), sex, country, and socio-economic status (SES). SES was derived from separate measures of income and education that were classified into within country tertiles (Low, Moderate, High). The mean of income and education was used to estimate a 3-level composite SES variable. Low SES corresponds to a low-low combination of income and education, and high SES corresponds to moderate-high and high-high combinations. Moderate SES corresponds to all other combinations of income and education. Where respondents refused to give their income (n = 1703), only education was used to estimate SES.

##### Tobacco Dependence

Dependence was assessed using the Heaviness of Smoking Index, (HSI) [26]. The HSI (range 0 - 6) was created as the sum of two categorical measures: number of cigarettes smoked per day (coded: 0: 0-10 cigarettes per day (CPD), 1: 11-20 CPD, 2: 21-30 CPD, 3: 31+ CPD), and time to first cigarette (coded: 0: 61+min, 1: 31-60 min, 2: 6-30 min, 3: 5 min or less). The HSI was then recoded into three categories of dependence: Low: 0 to 1, Moderate: 2 to 3, and High: 4 to 6.

### Analysis

Bivariate correlations were performed to explore associations between different belief measures. Chi-square tests were used to examine country differences in reported past year use of SLT and stop-smoking medications at each wave. A separate multivariate analysis was run for each of the four beliefs to determine whether there were overall (i.e. collapsed across waves) differences by country and each of the other covariates. In order to control for the correlations between responses from respondents who had data on multiple wave-to-wave transitions, the multivariate models were tested using a Generalised Estimating Equation (GEE) [27] with binomial variations, logit link function and an unstructured correlation structure. To explore whether there was a systematic longitudinal trend in each belief, survey wave was included. All variables were entered as a categorical variable, except survey wave which was treated as a continuous variable. We subsequently tested interactions between survey wave and country, and between country and the sociodemographic variables. Only significant interactions will be discussed in the results. All reported frequencies and analyses are based on weighted data to control for sampling and attrition biases due to age, sex, and geographic region. Statistical significance is set to p < .05. All analyses were performed using Stata v.10.

## Results

Table 2 shows the usage of stop-smoking medications and SLT in the four countries at each wave. Use of NRT declined after wave 5 in Canada, the UK, and Australia and after wave 6 in the US. Use of any SSMs increased up to wave 5 (2006) but then may have stabilised, indicating an increased use of prescription-only medications in the last two waves. NRT use remains the strongest in the UK (p < .001). SLT use was most common in the USA, and there were no trends over time (p > .05 in all countries).

**Table 2:** Proportion of respondents reporting use of smokeless tobacco, any stop-smoking medication, and nicotine replacement therapy within each country (%; weighted)

	2002	2003	2004	2005	2006	2007	2008
Used SLT in last 12 months							
Canada	2.5	1.3	2.3	2.3	2.0	2.4	2.3
US	6.4	4.6	5.5	6.8	6.0	7.6	6.2
UK	2.2	1.6	1.7	1.7	1.9	2.1	1.9
Australia	1.9	1.0	1.7	1.0	1.6	1.0	1.4
χ^2 ^	χ^2 ^= 93.5**	χ^2 ^= 77.2**	χ^2 ^= 68.6**	χ^2 ^= 127.7**	χ^2 ^= 83.7**	χ^2 ^= 145.8**	χ^2 ^= 73.7**
Used any SSM in last 12 months							
Canada	16.5	15.4	18.7	18.1	20.3	21.5	22.0
US	11.9	10.5	12.2	16.5	15.5	20.5	21.4
UK	12.7	11.8	16.7	22.2	23.2	21.3	21.6
Australia	16.1	14.1	16.6	18.2	22.3	22.3	22.0
χ^2 ^	χ^2 ^= 28.9**	χ^2 ^= 25.2**	χ^2 ^= 31.4**	χ^2 ^= 20.0**	χ^2 ^= 38.2**	χ^2 ^= 1.7	χ^2 ^= 0.2
Used NRT in last 12 months							
Canada	13.1	11.9	15.4	15.9	17.8	17.1	13.4
US	8.8	7.4	9.0	12.7	12.6	13.1	11.0
UK	11.7	11.3	15.8	21.4	22.4	19.2	17.8
Australia	13.4	12.3	14.8	15.9	20.0	18.9	15.6
χ^2 ^	χ^2 ^= 27.8**	χ^2 ^= 30.1**	χ^2 ^= 50.2**	χ^2 ^= 50.1**	χ^2 ^= 61.6**	χ^2 ^= 28.7**	χ^2 ^= 30.8**

NB: SLT = Smokeless tobacco, ST = Smoked tobacco, & NRT = Nicotine replacement therapy, & SSM = Stop smoking medication. ** = p < 0.01.

Overall, there were low correlations, all in the expected direction, between each of the beliefs suggesting some inconsistency in respondents' knowledge about the safety of SLT or nicotine alternatives (see Table 3). The strongest association was the belief that NRT is a lot less harmful than smoked tobacco being positively associated (as expected) with disagreeing that stop-smoking medication might be harmful to health. We looked for any notable change in the strength of these associations across waves but found none, nor was there any systematic difference in these correlations between countries.

**Table 3:** Correlations among beliefs (range across waves: lowest correlation to highest correlation)

	SLT is less harmful than ST^	SSM is not harmful to health	Nicotine does not cause most cancer	NRT is a lot less harmful than cigarettes
SLT is less harmful than ST	1.00	--	--	--
SSM is not harmful to health	0.032* to 0.115**	1.00	--	--
Nicotine does not causes most cancer	0.041** to 0.072**	0.000 to 0.017	1.00	
NRT is a lot less harmful than cigarettes	0.171** to 0.209**	0.248** to 0.265**	0.140** to 0.148**	1.00

NB: SLT = Smokeless tobacco, ST = Smoked tobacco, & NRT = Nicotine replacement therapy, & SSM = Stop smoking medication. * = p < 0.05 & ** = p < 0.01. ^ Among smokers aware of SLT.

### Belief that nicotine is not the chemical that causes most of the cancer

**Figure 1:**
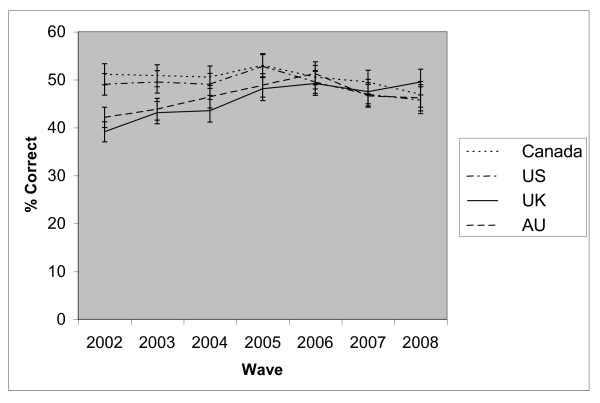
**The proportion of respondents who correctly reported that nicotine is **not **the chemical in cigarettes that causes most of the cancer, by country**.

Whilst respondents in the UK were least likely to report that nicotine is not the chemical that causes most of the cancer between waves 1 and 4, from wave 5 the difference between countries was not significant (see Figure 1). The interaction between survey wave (treated as a linear variable) and country was significant (p < 0.001). Correctly reporting that nicotine is not the chemical that causes most of the cancer significantly declined in Canada (OR = 0.98, p = 0.021) and the US (OR = 0.97, p = 0.002), whilst significantly increasing in the UK (OR = 1.05, p < 0.001) and Australia (OR = 1.02, p = 0.030). Overall, males, those of higher SES, younger respondents, those higher on the HSI, and those who had used any SSM (but curiously not NRT alone), or had used SLT in the past year were more likely to hold this belief (see Table 4).

**Table 4:** Predictors of beliefs about tobacco alternatives (GEE analyses; data weighted)

	NRT is a lot less harmful than smoking cigarettes		SLT is less harmful than smoked tobacco*		SSM is not harmful to health		Nicotine does not cause most of the cancer	
	
Number of observations	33, 534		27,149		50,004		50,147	
	
	OR	95% CI	OR	95% CI	OR	95% CI	OR	95% CI
Survey wave (continuous)	**1.06**	**1.04 - 1.08**	**1.05**	**1.03 - 1.07**	**1.04**	**1.02 - 1.05**	1.00	0.99 - 1.01
Country								
UK	1.00		1.00		1.00		1.00	
Canada	**0.55**	**0.50 - 0.60**	**0.41**	**0.37 - 0.46**	**0.59**	**0.55 - 0.63**	**1.18**	**1.09 - 1.27**
US	**0.48**	**0.44 - 0.53**	**0.31**	**0.28 - 0.35**	**0.57**	**0.53 - 0.62**	**1.16**	**1.08 - 1.25**
Australia	**0.66**	**0.60 - 0.72**	**0.80**	**0.72 - 0.89**	**0.69**	**0.65 - 0.75**	1.03	0.95 - 1.11
Gender								
Female	1.00		1.00		1.00		1.00	
Male	**1.13**	**1.06 - 1.21**	**1.17**	**1.08 - 1.27**	**1.06**	**1.01 - 1.12**	**1.31**	**1.24 - 1.38**
Age								
18 to 24	1.00		1.00		1.00		1.00	
24 to 39	0.93	0.82 - 1.06	**0.72**	**0.63 - 0.83**	1.00	0.91 - 1.10	**0.81**	**0.73 - 0.89**
40 to 54	0.92	0.81 - 1.04	**0.73**	**0.63 - 0.83**	1.08	0.99 - 1.18	**0.60**	**0.55 - 0.66**
55+	**0.67**	**0.59 - 0.76**	**0.63**	**0.54 - 0.74**	0.91	0.83 - 1.01	**0.49**	**0.44 - 0.54**
SES								
Low	1.00		1.00		1.00		1.00	
Moderate	**1.38**	**1.28 - 1.49**	**1.15**	**1.04 - 1.28**	**1.12**	**1.06 - 1.19**	**1.40**	**1.31 - 1.49**
High	**2.01**	**1.83 - 2.20**	**1.34**	**1.19 - 1.51**	**1.11**	**1.03 - 1.19**	**2.38**	**2.21 - 2.58**
HSI								
Low	1.00		1.00		1.00		1.00	
Moderate	1.02	0.95 - 1.10	1.08	0.98 - 1.18	**1.10**	**1.04 - 1.16**	1.03	0.97 - 1.08
High	1.07	0.98 - 1.16	1.07	0.97 - 1.19	**1.11**	**1.05 - 1.19**	**1.11**	**1.03 - 1.17**
Used NRT in past year								
No		1.00		1.00	1.00			1.00
Yes	**1.26**	**1.10 - 1.46**	1.03	0.85 - 1.24	**1.13**	**1.00 - 1.27**	0.97	0.87 - 1.09
Used SLT in past year								
No		1.00		1.00	1.00			1.00
Yes	1.09	0.92 - 1.30	**1.80**	**1.51 - 2.14**	**0.84**	**0.74 - 0.97**	**1.27**	**1.11 - 1.44**
Used any SSM in past year								
No		1.00		1.00	1.00			1.00
Yes	**1.37**	**1.21 - 1.56**	1.01	0.84 - 1.20	1.10	0.98 - 1.22	**1.13**	**1.02 - 1.26**

NB: Odds ratios are after controlling for all variables shown in the table, and survey wave

* Analysis includes only smokers aware of SLT. CI = confidence interval. Bold text indicates p < .005.

### Beliefs about the safety of stop-smoking medications

**Figure 2:**
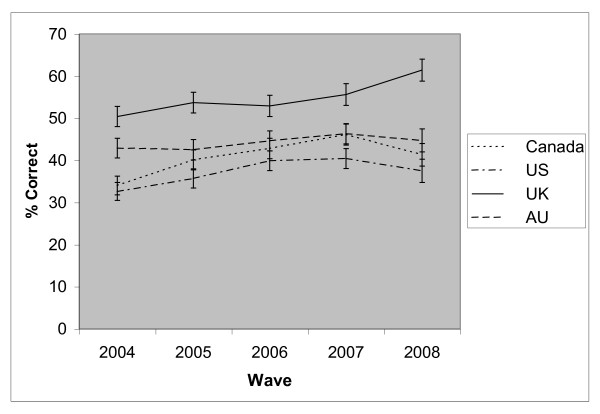
**The proportion of respondents who correctly reported that nicotine replacement therapy is a **lot less **harmful than smoking cigarettes, by country**.

Compared to respondents in Canada, the US and Australia, respondents in the UK were more likely to report that NRT is a lot less harmful than smoked tobacco at each wave (see Figure 2). Overall, males, those of higher SES, and those who had used NRT (or indeed any SSM) in the past year were more likely to hold this belief (see Table 4). The interaction between survey wave and country was significant (p = 0.048). This belief increased significantly in Canada (OR = 1.09, p < 0.001), the US (OR = 1.05, p = 0.007), and the UK (OR = 1.08, p < 0.001), but not Australia (OR = 1.02, p = 0.195). The interaction between country and gender was also significant (p = 0.011). Males were significantly more likely than females to hold this belief in Canada (OR = 1.32, p < 0.001) and Australia (OR = 1.16, p = 0.022), but not in the US (OR = 1.05, p > 0.05) or the UK (OR = 1.02, p > 0.05).

**Figure 3:**
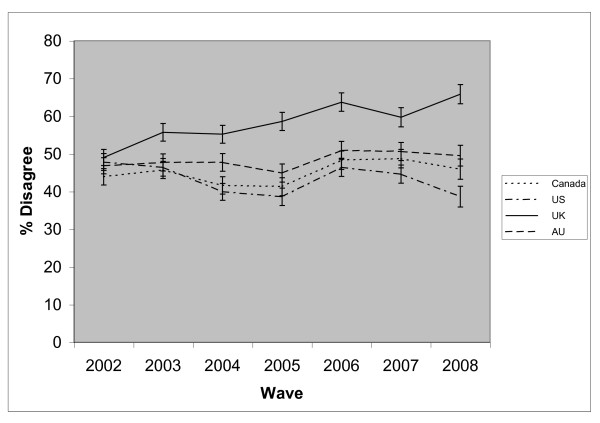
**The proportion of respondents who **disagree **that that stop-smoking medications might be harmful to health, by country**.

At each wave, respondents in the UK were the most likely to disagree that stop-smoking medications might be harmful to health (see Figure 3). The interaction between survey wave and country was significant (p < 0.001). The proportion of respondents disagreeing that stop-smoking medications might harm health significantly increased in Canada (OR = 1.03, 95% CI = 1.01 - 1.05), the UK (OR = 1.11, 95% CI = 1.09 - 1.13) and Australia (OR = 1.03, 95% CI = 1.01 - 1.06) whilst significantly decreasing amongst US respondents (0.96, 95% CI = 0.94 - 0.99). Overall, males, those of moderate to high SES and HSI, and those who had used NRT in the past year, were more likely to hold this belief (see Table 4). Significant interactions were also found between country and age group (p < 0.001), SES (p = 0.003), and gender (p = 0.003). There was no significant age effect in Canada or Australia. In the US, those aged 40 to 54 were significantly more likely to hold this belief than 18 to 24 year olds (OR = 1.22, 95% CI = 1.01 - 1.47). In the UK, those aged over 55 were significantly less likely than 18 to 24 year olds to hold this belief (OR = 0.61, 95% CI = 0.49 - 0.75). Compared to low SES, high SES smokers were more likely to hold this belief in Canada (OR = 1.19, 95% CI = 1.02 - 1.38) and the US (1.22, 95% CI = 1.06 - 1.41), whilst moderate SES smokers were more likely in the UK (1.15, 95% CI = 1.02 - 1.30). There was no SES effect in Australia. A significant gender effect was found only in Canada where men were significantly more likely to hold this belief than women (OR = 1.24, 95% CI = 1.12 - 1.37).

### Belief about the safety of smokeless tobacco

Reported awareness of SLT products was highest in the US at each wave (mean proportion = 82.3%) and lowest in the UK (mean proportion = 52.7%). The mean proportion of smokers aware of SLT in Canada and Australia was 72.9% and 61.1%, respectively. Being aware of SLT was associated with being male, high SES, and aged 39 or under.

**Figure 4:**
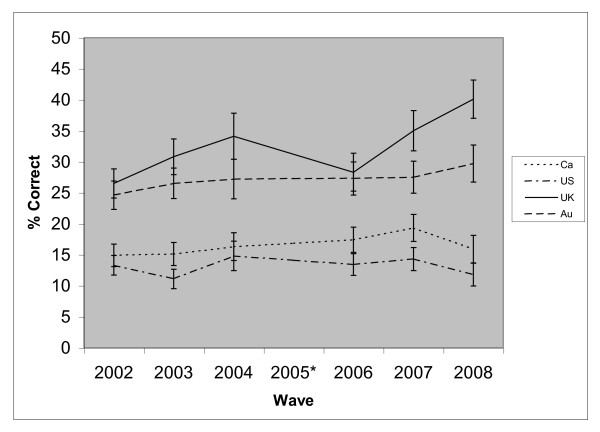
**The proportion of respondents (aware of SLT) who correctly reported that there are forms of SLT **less **harmful than smoked tobacco, by country**. *Question not included at wave 4.

Among those aware of SLT, reporting that there are forms of SLT less harmful than smoked tobacco was highest in the UK at each wave, although not significantly different from Australia between waves 1 and 5 (see Figure 4). Between wave 5 and 7, the proportion in the UK increased from 28.3 to 40.1 compared to only 27.3 to 29.7 in Australia. The interaction between survey wave and country was significant (p = 0.001). The proportion in Canada and the US did not significantly change, whilst it significantly increased in the UK (OR = 1.10, p < 0.001) and Australia (OR = 1.05, p = 0.014). Overall, males, younger respondents, those of higher SES, and those who had used SLT in the past year were more likely to hold this belief (Table 4). The interaction between country and gender was significant (p = 0.001). Males were significantly more likely than females to hold this belief in Canada (OR = 1.27, p = 0.004) and the US (OR = 1.55, p < 0.001), but not in the UK (OR = 1.04, p > 0.05) or Australia (OR = 1.02, p > 0.05). The interaction between country and age group was also significant (p = 0.007). Respondents aged 18 to 24 years old were significantly most likely to hold this belief in the UK and Australia only. The age variable was not a significant predictor in Canada or the US.

Despite an improvement in this belief among smokers who were aware of SLT, among all smokers (i.e. regardless of awareness) this knowledge showed no significant linear improvement in any of the four countries. In the UK, there was a significant increase between wave 6 and 7 (17.4% at wave 6 to 26.3% at wave 7). This was primarily due to increased awareness of SLT in the UK between waves 6 and 7 (49.5% to 64.9%).

From wave 3, smokers who were aware of SLT and reported that it was less harmful than smoked tobacco were asked whether it was a little or a lot less harmful. As a proportion of smokers aware of SLT, there was no significant improvement in the knowledge that some forms of SLT are a lot less harmful than smoked tobacco in any of the four countries. Overall, UK smokers were significantly more likely to report that SLT is a lot less harmful than smokers in Canada (OR = 3.33, 95% CI = 2.71 - 4.08), the US (OR = 5.20, 95% CI = 4.20 - 6.43), and Australia (OR = 1.42, 95% CI = 1.19 - 1.68).

Table 4 presents the results of the GEE analyses for each of the four beliefs, showing the main effects for country and sociodemographic factors. Overall, respondents who were better informed about the safety of NRT and SLT relative to smoked tobacco were more likely to be aged 18 to 24, male and of high SES. The same demographic profile was found for respondents who agreed that nicotine is not the chemical that causes most of the cancer. Respondents who disagreed that stop-smoking medications might be harmful to health were more likely to be of moderate to high SES and there were varying associations with age across the four countries. We found no evidence to suggest that the UK's overall better knowledge about the safety of alternatives to smoked tobacco was confined to any particular sociodemographic group.

## Discussion

Knowledge about the relative harmfulness of tobacco products and nicotine remains low and the situation is worse among those of low SES and, in most cases female smokers. In late 2008, only about a half of smokers correctly reported that nicotine is not the chemical in cigarettes that causes cancer and the proportion having this correct belief had only increased in recent years in the UK and Australia. However, in Australia, this was not matched by an increase in the belief that NRT is a lot less harmful than cigarettes, which increased in the three other countries.

In Canada and the US where SLT is legally available, only around one in six smokers believed that some SLT products could be less harmful than cigarettes. No noticeable change over the seven years of study suggests that this perception is entrenched in the minds of most smokers. It is somewhat intriguing that smokers in the UK and Australia, countries where most SLT products are banned, appeared to be better informed about the relative health risks of SLT compared to cigarettes, with around a quarter at the outset believing that some SLT products could be less harmful than cigarettes. In these countries, having this correct belief increased over the lifetime of the study, with the biggest increase occurring between 2006 and 2009 in the UK. However, in 2008-2009 this view was only held by a minority of smokers surveyed (40%) in the UK.

The only country in our study where there was consistent evidence of improving knowledge about the relative health dangers of smoking to alternative forms of nicotine delivery was in the UK where significant efforts have been made over the past decade to promote the use of NRT as a substitute for cigarettes.

The main strength of this study is the broadly representative nature of the sample of smokers in each country, coupled with the capacity to weight the data to improve the accuracy of estimates. The main weakness is that this study only recruited cigarette smokers so users of other tobacco products are not represented unless they also smoke cigarettes. Thus, this study has nothing to say about the views of other tobacco product users in general.

The finding that each of the four beliefs we studied, although logically related given the evidence, were largely independent of one another suggests there is a low level of real understanding among smokers, even among those who 'know' some of the correct answers. This is an important gap in knowledge with potential adverse public health implications if it leads to under-use of NRT and other medications, or if it leads to continued use of cigarettes instead of seeking out harm-reducing alternatives. Further research is required to explore whether misinformation is a deterrent to using alternatives to smoked tobacco. The entrenched incorrect beliefs in North American smokers suggest that mere availability of the products with the attendant commercial activity encouraging their use is insufficient to produce adequate consumer knowledge. Regardless, governments have a responsibility to ensure that something is done. We suspect that part of the problem is that smokers are generalising from their knowledge of cigarettes to assume all tobacco products, indeed anything to do with tobacco, is seen as bad. Manufacturers of these products have clearly failed to educate consumers about the relative health benefits of using alternative forms of tobacco compared to cigarettes. Whether we should expect them to improve their consumer education or have government take over this role is unclear, and may vary by jurisdiction. With the advent of FDA regulation of tobacco products in the USA, including a mechanism for approval to market products as 'modified-risk', and evidence for growth of the SLT category, the opportunity may present itself in the near future to provide the kind of public education that is so clearly needed. Other countries will need to develop comparable mechanisms.

In conclusion, smokers remain misinformed about the relative safety of nicotine and tobacco products, though some hopeful signs for improvement are evident in the UK, where there have been concerted efforts to educate health professionals and through them, the public, about stop smoking medications.

## Authors' contributions

RB conceived of the study, drafted parts of the original draft and supervised all aspects. JC conducted the statistical analysis and drafted sections of the manuscript. RB, AM, RO'C, and KMC participated in the design of the study and the interpretation of the results. All authors participated in revising the manuscript, and read and approved the final manuscript.

## Competing interests

The authors declare that they have no competing interests.
